# Evolutionary change in the human gut microbiome: From a static to a dynamic view

**DOI:** 10.1371/journal.pbio.3000126

**Published:** 2019-02-07

**Authors:** Isabel Gordo

**Affiliations:** Instituto Gulbenkian de Ciência, Oeiras, Portugal

## Abstract

Our intestine is a melting pot of interactions between microbial and human cells. This gene-rich ecosystem modulates our health, but questions remain unanswered regarding its genetic structure, such as, “How rapid is evolutionary change in the human gut microbiome? How can its function be maintained?” Much research on the microbiome has characterized the species it contains. Yet the high growth rate and large population sizes of many species, and the mutation rate of most microbes (approximately 10^−3^ per genome per generation), could imply that evolution might be happening in our gut along our lifetime. In support of this view, Garud and colleagues present an analysis that begins to unravel the pattern of short- and long-term evolution of dozens of gut species. Even with limited longitudinal short-read sequence data, significant evolutionary dynamics—shaped by both positive and negative selection—can be detected on human microbiomes. This may only be the tip of the iceberg, as recent work on mice suggests, and its full extent should be revealed with dense time series long-read sequence data and new eco-evolutionary theory.

## Abstract 2

Trillions of bacteria inhabiting our intestinal tract contribute to our wellbeing. This Primer explores how the known capacity of bacteria to rapidly adapt in the lab plays out in our own bodies; evolutionary changes are detected within months, allowing commensal bacteria to evolve through our lifetime.

## Introduction

The intestine of every human is home to an extremely rich microbial ecosystem. Understanding its diversity, stability, and resilience and how these influence health is a remarkably hard problem. At a scale of millions of years, as comparative genomics reveals, microbiota composition has been marked by a long-term co-evolutionary history between microbes and their primate hosts [1]. At a much smaller scale (i.e., weeks), ecological processes such as nutritional changes or external perturbations change microbiota species composition in the human gut. For example, strong and sustained dietary changes have been shown to shape the abundance of different species in both mice and humans [2,3]. Treatment with some antibiotics has also been shown to cause reductions in microbiota species diversity, with potential increases in microbe transmission between individuals [4]. Many studies have therefore focused on characterizing how these and other factors associated with disease conditions may alter species abundance (microbiota composition, estimated by 16S rRNA sequencing) or the abundance of microbial genes (microbiome, estimated by shotgun sequencing) within the gut [5]. However, the process of evolutionary change within each species during the lifetime of a given host is far less understood. Imagine that Charles Darwin was here today and we gave him a population genetics textbook [6] and a next generation sequencer. Would he go on another trip on the Beagle, or stay at home and study his gut microbes to find the molecular signatures of his theory of natural selection?

Evolution in the microbiome via natural selection on standing genetic variation, on new lineages formed by recombination, or on de novo mutation, has been vastly neglected. Yet it is the understanding of mechanisms shaping evolution at these microscales (i.e., population genetics) that ultimately allows explaining macroscale observations. Such knowledge and the constant confrontation of data with the predictions of population genetics theory, either to reject or support, will permit correct inferences of the causes responsible for the levels of bacterial diversity currently observed in the human species.

## Evolutionary change of microbes in the gut?

Mutation is the only process capable of originating diversity that does not yet exist. However, because most mutations are deleterious, the spread of new mutations to detectable frequencies within a host microbiome could be a rare event. A back-of-the-envelope calculation may nevertheless indicate otherwise. The typical census population size of a commensal species of the human gut should easily approach 10^7^ to 10^8^ cells/g (e.g., *Escherichia coli*, which is one of low abundance). If the mutation rate in the gut is similar to estimates in the laboratory (i.e., approximately 10^−3^ per genome per generation [7]), then in each gram of material there will be 10^4^ to 10^5^ new mutant cells. Of these, only a fraction would carry alleles with local benefits to its bacterial host as it competes in the gut ecosystem. If this fraction is minute (i.e., 1 in 1 million mutations is of any good at any moment), then evolutionary change via this mechanism will be very slow. But what if that fraction is similar to that observed in laboratory experiments (e.g., 1 in 100 or 1,000 [8,9])? That would result in the production of hundreds of mutant clones with potential to spread within the species in a blink. It would turn the time scale of evolution from years to days, as has been seen in gut bacteria colonizing mice [10]. Can such a process be occurring within the microbiome of every human every month? If so, one should be able to “see” the rapid increase in frequency of single nucleotide variants (SNVs), as is expected when a beneficial mutation spreads and sweeps in a population. A similar reasoning applies regarding the process of recombination. The ability of bacteria to shuffle genes by recombination in the gut is likely to be higher than typical estimates in the lab or other environments [11,12].

## Searching for evolution in the human gut microbiome

In this issue, Garud and colleagues [13] seek to find signatures of evolutionary events and the action of natural selection. They use time series data of the Human Microbiome Project and also microbiome data of twins. The first allows comparing two samples of the same host and search for evolutionary changes in a lineage that is colonizing that host during a time scale of months. The second should identify potential evolutionary change at the scale of years. The short reads of the current microbiome data would a priori deem this a daunting task. With short reads, it is extremely difficult to tell apart which mutation is in what genome, which makes it difficult to find de novo mutation, or which SNV is linked to another SNV (i.e., in the same genome), essential to determine levels of genetic association—linkage disequilibrium—and to measure recombination. The natural process of host colonization at birth is idiosyncratic, sometimes involving a single lineage of a given species, sometimes involving a handful of lineages (i.e., oligo-colonization). This variation creates further difficulties in detecting evolutionary change. Through using population genetics theory as background and advancing on previously developed methods for metagenome analysis [14,15], Garud and colleagues provide estimates of the strength of both purifying and positive selection likely to shape human microbiomes, at different time scales.

In moving forward to dissect the evolutionary mechanisms and their strength along time, Garud and colleagues take a radically new approach: do not focus on what is inside a single host, focus instead on a large panel of hosts that are colonized by the same species. Within some hosts, some species will have a simple lineage structure that is amenable to estimate how much evolution it experiences. For other species, other hosts will have that property. Indeed, Garud and colleagues’ insight was to focus on a given species and scrutinize only the easiest host samples, i.e., for which a dominant lineage of that species can be identified with high statistical confidence. This comes at a trade-off of discarding many host samples, because the level of complexity of the populations that colonized them would forbid estimation of linkage between SNVs (i.e., determining haplotypes). Nevertheless, this procedure did allow straightening out three key issues—the limited role of host relatedness at explaining major patterns of the population genetic structure of gut microbes, the rejection of neutrality in shaping SNV frequencies and linkage levels between SNVs, and the tip of the iceberg on how much evolution via de novo mutation and/or recombination is happening inside a human.

The observed patterns of between-host polymorphism reject the predictions of a simple neutral model of molecular evolution for several human gut bacteria. Synonymous site polymorphism (i.e., that does not lead to changes in the protein sequence) exhibits a variance clearly inconsistent with a model, in which neutral mutations arise in each host and a single lineage transmits between hosts. However, the pattern of polymorphism at synonymous and nonsynonymous sites is consistent with the slightly deleterious theory of molecular evolution [16], in which widespread purifying selection may keep a microbial ecosystem functional, at long time scales, for all hosts. Much of the variation observed can be explained by postulating the recurrence of a high fraction (90%) of mutations whose effects decrease fitness by a very modest amount (approximately 0.01%) but still strong enough to be purified in the long run.

When looking for signs of evolution (identity by descent with modification) within hosts, significant changes in SNV frequency could be detected on a time scale of six months (occurring in 12% of the time comparisons). This was possible even under the strong criteria imposed for low false-positive rates (frequency changes above 60%), which can greatly limit power to detect true events that may occur but remain undetected with this type of data. Of the identified events, approximately three-fourths involved a handful of SNVs rising to high frequency on a time scale of hundreds of generations. Such an observation is highly unlikely under neutral evolution (in which mutations would take a much longer time to change in frequency) but fully consistent with natural selection increasing the frequency of mutation and/or recombination created alleles having fitness effects of a few percent. The remaining one-fourth of the detected changes involved thousands of SNVs, compatible with the replacement of a dominant strain by a newcomer invader strain or with a rapid spread of another resident strain, which was colonizing that host at low frequency [14]. Furthermore, albeit under the strong filtering criteria imposed to avoid false negatives and/or positives, gene content differences could also be inferred. These involved gene losses (caused by mutation [deletions] or recombination) and gene gains, potentially recombination derived, which tended to change the accessory (noncore) genome.

What consequences should these short-term evolution dynamics have? It could lead to local host adaptation and diminish the chances of invasion of the gut of that host by external strains. To address this, Garud and colleagues look at the microbiomes of adult twins. If these are initially colonized by similar strains, they can serve as a proxy for how much evolution can happen after decades of exposure to the global environment. The comparison of SNVs in the twins’ samples revealed that replacement by external strains was the dominant process, indicating that, whatever form of host-specific selection occurs in the short term [17], it may not last in the long term.

## Gut microbial evolution into the future

The analysis of Garud and colleagues suggests a new conceptual model for human gut microbes and ways to test it. In such a model, the interplay of de novo mutation, recombination, and strong positive selection occurring within hosts, which creates personalized and dynamic diversity in months, is swamped by between-host migration of strains on a time scale of decades. Their observations cry out for dense longitudinal sampling of human gut microbiomes in the future. Such a sampling effort will also be important to advance from studies in which microbiome diversity is associated with specific factors, towards studies in which the causes of that diversity can be unraveled [5]. Their population genetics framework of successively rejecting simple neutral models demands novel theory in which all the mechanisms generating variation by creation, reshuffling, and transmission work along with a complex form of selection to result in highly dynamic and functional microbiomes.

## Abbreviation

SNV: single nucleotide variant
